# Human genetics as a model for target validation: finding new therapies for diabetes

**DOI:** 10.1007/s00125-017-4270-y

**Published:** 2017-04-26

**Authors:** Soren K. Thomsen, Anna L. Gloyn

**Affiliations:** 1 Oxford Centre for Diabetes, Endocrinology and Metabolism, University of Oxford, Churchill Hospital, Old Road, Headington, Oxford, OX3 7LE UK; 2 Wellcome Trust Centre for Human Genetics, University of Oxford, Oxford, UK; 3 National Institute of Health Research Oxford Biomedical Research Centre, Churchill Hospital, Oxford, UK

**Keywords:** Adverse effects, Genome-wide association studies, Human genetics, Monogenic diabetes, Precision medicine, Review, Target discovery, Target validation, Therapeutic mechanisms, Type 2 diabetes

## Abstract

Type 2 diabetes is a global epidemic with major effects on healthcare expenditure and quality of life. Currently available treatments are inadequate for the prevention of comorbidities, yet progress towards new therapies remains slow. A major barrier is the insufficiency of traditional preclinical models for predicting drug efficacy and safety. Human genetics offers a complementary model to assess causal mechanisms for target validation. Genetic perturbations are ‘experiments of nature’ that provide a uniquely relevant window into the long-term effects of modulating specific targets. Here, we show that genetic discoveries over the past decades have accurately predicted (now known) therapeutic mechanisms for type 2 diabetes. These findings highlight the potential for use of human genetic variation for prospective target validation, and establish a framework for future applications. Studies into rare, monogenic forms of diabetes have also provided proof-of-principle for precision medicine, and the applicability of this paradigm to complex disease is discussed. Finally, we highlight some of the limitations that are relevant to the use of genome-wide association studies (GWAS) in the search for new therapies for diabetes. A key outstanding challenge is the translation of GWAS signals into disease biology and we outline possible solutions for tackling this experimental bottleneck.

**Electronic supplementary material:**

The online version of this article (doi:10.1007/s00125-017-4270-y) contains a slideset of the figures for download, which is available to authorised users.

## Introduction

Long-term complications of type 2 diabetes and its related disorders present a major, growing socioeconomic burden to society [1]. Despite incremental advances in the development of therapies for diabetes, current treatments fail to provide adequate glucose control for most patients. Much-needed efforts to develop novel, first-in-class drugs are hampered by the slow rate and escalating costs of research and development programmes in the pharmaceutical industry. The staggering estimated price tag for an average new drug is approaching $3 billion, with the high attrition rate in clinical trials (>80%) imposing a cumulatively higher cost on those drugs that do make it to market [2, 3]. The most common reasons for failure include lack of efficacy and/or unsuitable safety profiles, even in cases where the correct target is engaged [4, 5]. Clearly, these observations attest to the limitations of existing preclinical models in evaluating therapeutic candidates before committing to expensive human studies.

Over the past decades, technological advances have unlocked the possibility of using human genetics as a complimentary strategy for preclinical target validation. Genetic variation offers valuable insights into the effects of manipulating specific proteins or pathways in a system that is directly relevant to human disease. Such ‘experiments of nature’ can, in principle, inform target identification, predict potential adverse long-term effects and identify suitable indications for treatment. In this review, we first discuss evidence for the benefits provided by human genetics for the treatment of diabetes within each of these three domains. Second, we focus on one of the key challenges facing this paradigm, specifically the identification of causal mechanisms for genetic variants, and provide examples of potential solutions.
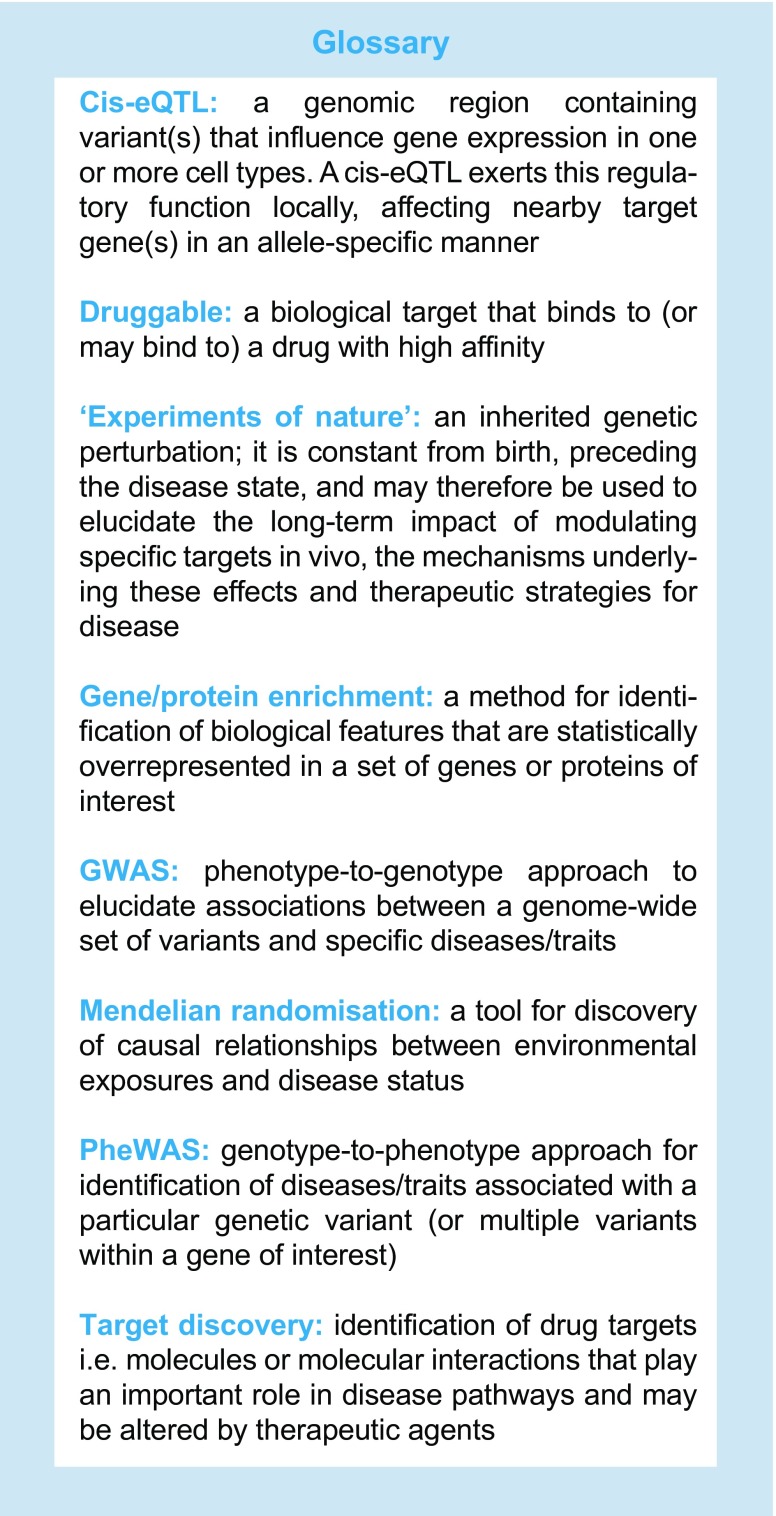


## ‘Experiments of nature’ in drug target discovery

**Fig. 1:**
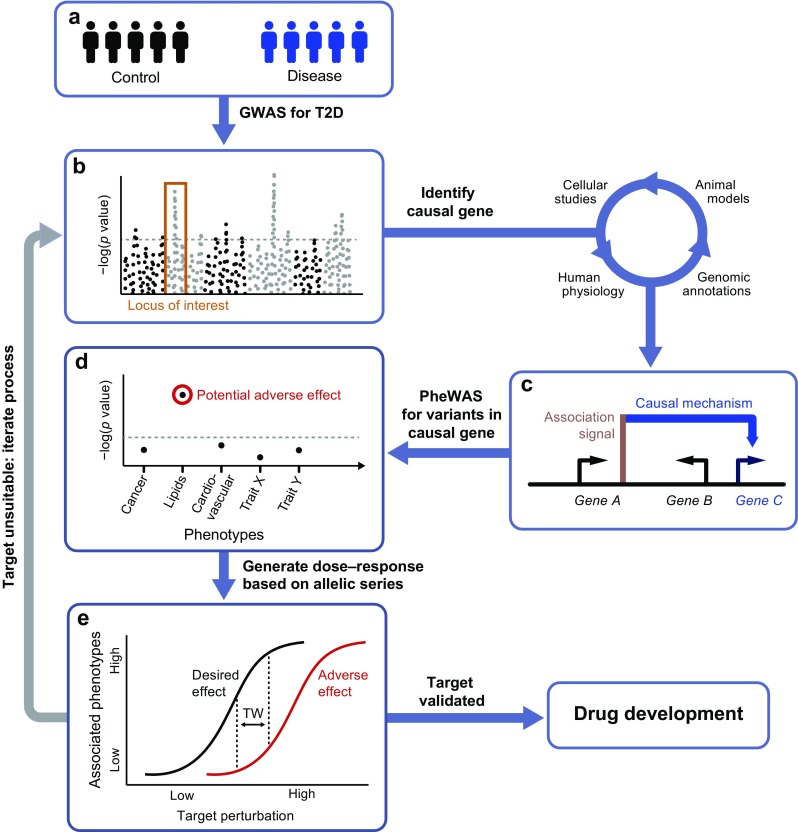
Using human genetics as a model for drug target validation. GWAS into the heritability of type 2 diabetes (T2D) have identified a large number of variants that are robustly associated with disease risk (**a**, **b**). Nevertheless, establishing the underlying causal mechanisms has proven to be a major experimental bottleneck. The process usually involves an array of approaches, including in vitro and in vivo studies in animal and cellular models, as well as genetic and physiological follow-up studies of risk-allele carriers. Once a causal gene has been identified (**c**), the encoded protein may be taken forward for further validation as a potential drug target. Genetic alleles within the causal gene can be interrogated for links to other phenotypes using PheWAS, which can highlight likely adverse or beneficial effects of long-term treatment (**d**). For candidate genes harbouring multiple, independent alleles, effects on disease risk can be correlated against their known impact on protein function (**e**). Some perturbations, such as protein-truncating variants, have predictable effects, while most alleles require extensive experimental follow-up to reliably ascertain their functional impacts. If an allelic series has been established, their phenotypic associations can be used to generate the genetic equivalent of a dose–response curve (**e**). The therapeutic window (TW) marks the range of perturbations that produce a suitable ratio between desirable effects (i.e. type 2 diabetes protection) and adverse effects (e.g. raised lipid levels). In cases where a potential treatment is not predicted to result in a net patient benefit, the target is considered unsuitable and the process can be repeated for a different candidate. However, if an appropriate TW has been identified, the target can be taken forward for drug development on the basis of this human genetic validation

Nomination of a therapeutic candidate for clinical testing is based on the expectation that modulating a specific target will result in a net benefit for patients, taking into account both desired and adverse effects. Supporting evidence is usually derived from extensive preclinical testing, including studies in animal and cellular models, as well as data from observational epidemiology [6]. Importantly, these sources are often unsuitable for establishing definitive proof of causality in humans, with the specific models either lacking direct relevance or being unable to confidently distinguish cause and effect [7]. In both of these areas, human genetics can complement existing lines of evidence with a relevant window into the chain of causality. A genetic perturbation (an inherited mutation in or near an affected protein) is constant from birth and thus precedes the disease state rather than being affected by the disease environment. Unlike other molecular phenotypes, such as metabolite or protein levels, genetic variants are, therefore, not prone to be confounded by reverse causation. Moreover, in well-mixed populations, genotypes are randomly assigned at conception, thus acting, in effect, as natural versions of a randomised controlled trial [8]. Taking advantage of these properties, genetic epidemiology can intersect with other types of preclinical evidence to establish a powerful framework for target discovery and validation (Fig. 1).

Though experiments of nature can be highly relevant tools for prioritising drug targets, a number of challenges limit the applicability of this strategy. First, many disease-causing variants are located in regions of the genome that do not code for proteins, making identification of causal mechanisms a substantial challenge (this is discussed in more detail later) [9]. Further, the impact of individual mutations on protein activity often cannot be determined a priori, which necessitates extensive in vitro work to establish both statistical associations and directions of effect [10]. Once causal mechanisms have been established, these insights can then be used to focus research and development efforts on producing a desirable therapeutic effect. For instance, if loss-of-function variants in a particular gene are linked to protective effects, modulating the encoded protein by antagonists would be an attractive target (and often a more tractable goal for medicinal chemists than protein activation).

Ultimately, genetic epidemiology is reliant upon the occurrence of natural variation (either single-nucleotide and/or structural variants) in genomic regions relevant to disease. Since the penetrance of disease-associated variants tends to be inversely related to their frequency in the population (because of negative selection), most common variants have small effect sizes, while more deleterious mutations are rare [11–13]. As a result, sequencing of large-scale case–control cohorts is required to establish associations across the frequency spectrum [12, 14]. Phenotypic selection and familial studies (e.g. focusing on extreme forms of diabetes) can enrich for high-penetrance mutations, but assigning pathogenicity and estimating effect sizes from such studies can be a challenge [10, 15, 16]. In the ideal scenario, multiple independent alleles with different degrees of effect on a phenotype can be used to calibrate a genetic ‘dose–response’ curve [7, 17]. Not only does this build confidence in a specific target but the allelic series can also be used to predict the magnitude of effect required to produce a therapeutic response in vivo (Fig. 1). It is worth noting, however, that not all mutations produce effects that can be linearly mapped to a simple dose–response curve. More subtle perturbations could, for example, lead to aspects of both gain- and loss-of-function.

### Genetic studies for the validation of drug mechanisms

Over the past 10 years, genome-wide association studies (GWAS) have made significant progress towards mapping the genetic heritability of many complex diseases [18]. In the context of drug development, however, these advances are, broadly speaking, too recent to assess their impact on prospective target discovery. Still, attempts can be made to validate the use of GWAS associations for target discovery based on existing drugs. One study looked at the proportion of drug mechanisms (defined as a target paired with its approved indication) supported by genetic evidence at the various stages of the drug development pipeline [19]. This proportion was found to increase from 2% at the preclinical stage to 8.2% for approved drugs, with the single largest increase occurring between phase II and phase III of clinical trials. Remarkably, based on such historical data, it was estimated that the rate of success was around twofold higher for a target–indication pair supported by GWAS or other human genetic data compared with pairs with no support. While any retrospective study will certainly have limitations (e.g. successful drug mechanisms might spur genetic research into particular targets), two observations support the overall conclusion of this study: first, the same correlation was found for GWAS data alone (success ratio: 1.8 for supported vs unsupported mechanisms), which is unlikely to be influenced by known biological mechanisms [19]; second, most potential confounding factors (e.g. unknown causal mechanisms for GWAS signals and incomplete mapping of genetic heritability) actually tend to bias observations towards the null hypothesis.

### Thiazolidinediones

For the treatment of type 2 diabetes, thiazolidinediones (TZDs) provide an instructive example of a drug mechanism that has been corroborated by genetic evidence since first being discovered. TZDs are a class of commonly used drugs that act primarily through activation of the peroxisome proliferator-activated receptor γ (PPARγ) to improve insulin sensitivity [20]. Within a few years after obtaining market approval in 1996, the gene encoding PPARγ (*PPARG*) was found to contain a missense variant (Pro12Ala) that associates with type 2 diabetes susceptibility [21–23]. Though the functional impact of this common variant remains uncertain, the subsequent discovery of rare, loss-of-function variants associated with disease risk have established direction-of-effect at this locus [24]. Genetic evidence thus points to a therapeutic benefit of PPARγ agonists, fully consistent with the clinical effects observed from TZDs. The Pro12Ala association has also since been replicated by GWAS for type 2 diabetes risk, despite the relatively small effect size of the risk allele (OR 1.16) [25]. As illustrated by this case, the measured effect size of a single genetic variant is not necessarily a useful predictor of therapeutic opportunities.

### Sulfonylureas

This lesson is further reinforced by insights from genetic studies on the ATP-sensitive potassium channel (K_ATP_), which couples glucose metabolism to insulin secretion in pancreatic beta cells. As early as 1942, sulfonylureas inhibiting the channel were found to display hypoglycaemic effects in animal studies [26]. Around 60 years later, genetic studies in humans identified a type 2 diabetes association signal that overlaps two genes, *KCNJ11* and *ABCC8*, which encode subunits of the K_ATP_ (OR 1.1–1.2) [27–29]. Subsequent molecular studies have confirmed the risk haplotype to produce a channel that is less sensitive to ATP inhibition, thus reducing insulin secretion [30]. In contrast, sulfonylureas promote closure of the channel to depolarise the beta cell and mobilise insulin granules [31]. Thus, these findings demonstrate how genetic discovery can successfully predict the therapeutic potential of a known target based on genetic variants with moderate effect.

### ZnT8 modulation

Although no validated drug targets have emerged from type 2 diabetes GWAS to date, recently identified coding variants have highlighted plausible candidates. One candidate that has been the focus of particular interest is the *SLC30A8* gene, encoding the zinc transporter 8 (ZnT8), which is expressed in insulin secretory granules. Initially, common risk variants of unknown functional importance had spurred commercial interest in the development of agonists, based on the assumption of a negative correlation between activity levels of ZnT8 and diabetes risk [32, 33]. This notion was challenged by a more recent study that focused on protein-truncating variants in *SLC30A8* to determine the effect of loss-of-function on type 2 diabetes susceptibility [34]. Strikingly, the study found that carriers that were haploinsufficient for ZnT8 were protected from type 2 diabetes, with a 65% reduction in disease risk. These observations provide strong evidence in favour of a therapeutic strategy based on ZnT8 inhibition. More broadly, this example also illustrates the value in considering use of an extended allelic series to more fully explore the effects of target modulation at various levels of inhibition and/or activation.

## Predicting adverse effects of new therapies

The suitability of a drug candidate is ultimately dependent on whether the therapeutic effect is expected to outweigh any on- and off-target adverse effects. These can be difficult to predict, especially if caused by unintended drug promiscuity, but attempts can be made to anticipate on-target side effects. In an analogous fashion to the use of GWAS for target identification, phenome-wide association studies (PheWAS) provide a tool for determining the long-term consequences of manipulating a target [35]. Rather than seeking to identify variants associated with a particular disease, a PheWAS is designed to systematically identify the diseases or traits associated with a particular variant (or multiple variants within a gene of interest). In the same way that genetic perturbations can pinpoint target proteins, the detected phenotypes are a consequence of life-long experiments of nature. Any pleiotropy thus raises the possibility of additional on-target effects from long-term target modulation (Fig. 1).

As for GWAS, there are a number of practical and conceptual limitations that apply to the PheWAS paradigm in the context of target validation. First, it is clear that the identified phenotypes (both therapeutic benefits and on-target adverse effects) may be restricted to a perturbation that is imposed over many years or is present at a specific stage of disease progression. For instance, in the case of type 1 diabetes, the identified association signals have primarily uncovered genes implicated in the immune system. Nevertheless, modulating immune function in individuals with type 1 diabetes is unlikely to be an effective therapy, since autoimmune beta cell destruction has already occurred. For such diseases, the therapeutic pathways for treating symptoms (e.g. insulin or beta cell replacement for type 1 diabetes) may be different from the susceptibility pathways (uncovered by genetics) that are relevant to preventing disease. More generally, the lifetime exposure of a genetic defect might also produce long-term secondary effects (e.g. through compensatory mechanisms) that are not directly predictive of acute therapeutic interventions.

A second limitation of PheWAS is the requirement for access to diverse, deeply phenotyped cohorts or electronic medical records with genotyping information. Though population-wide biobanks and large, industry-led cohorts with sequencing data are now taking form, systematic PheWAS have previously been impractical [35]. Studies of this nature have, therefore, been more akin to traditional candidate gene association studies, focusing on a specific hypothesis concerning a target gene and a selected outcome. Nonetheless, recent examples of this approach being applied to the development of new treatments for type 2 diabetes provide insights into the potential value of PheWAS.

### Glucokinase and glucokinase regulatory protein

Glucokinase (encoded by *GCK*) is a key glycolytic enzyme involved in sensing the energy status of the body’s major organs. The protein is regulated in the liver by glucokinase regulatory protein (GKRP; encoded by *GCKR*), which sequesters glucokinase during fasting [36]. Genetic variation in both *GCK* and *GCKR* has been implicated in type 2 diabetes susceptibility, and the proteins are both targets of ongoing drug development efforts to modulate this pathway [37–39]. While increasing glucokinase activity (e.g. through GKRP inhibition or allosteric activation) could lower plasma glucose to reduce the risk of type 2 diabetes, genetic evidence also points to the possibility of likely adverse effects [40–42]. Several studies of deleterious variants in *GCKR* have found increased risk of hypertriacylglycerolaemia, probably as a consequence of elevated substrate availability for hepatic lipogenesis [43–46]. Interestingly, in clinical trials of one glucokinase activator, mild dyslipidaemia was reported in treatment groups, providing preliminary confirmation of this potential adverse effect [47]. Similar results were reported across different classes of glucokinase activators in rodents, arguing for an effect that is independent of the specific chemical compound [48]. In light of corroborating genetic and molecular data, it is clear that monitoring lipid levels for therapies targeting glucokinase/GKRP is essential.

### Sodium–glucose cotransporter 2

In a similar way, genetic evidence has been able to shine light on the clinical use of sodium–glucose cotransporter 2 (SGLT2) inhibitors, an emerging class of glucose-lowering drugs that act through increased renal clearance of glucose [49]. A naturally occurring inhibitor of SGLT2 (phlorizin) had been known for some time, spurring the development of synthetic analogues for use in humans [50]. Nevertheless, the discovery that familial renal glycosuria is caused by genetic variants in the gene encoding SGLT2 (S*LC5A2*) provided an opportunity to test for any side effects of long-term perturbations [51, 52]. Individuals carrying loss-of-function alleles in S*LC5A2* have reduced ability to reabsorb glucose in the kidney but display otherwise normal renal function and no or few additional clinical features (www.omim.org/entry/233100, accessed 1 March 2017). These observations suggest that selective targeting of SGLT2, even for prolonged periods of time, is not associated with any significant complications.

### *PTEN*

Another rare genetic disorder, known as Cowden’s syndrome, has offered new clues into a possible link between type 2 diabetes, obesity and cancer, as initially suggested by epidemiological data [53]. The majority of patients suffering from the cancer predisposition syndrome carry germline loss-of-function mutations in the *PTEN* gene [54]. The protein encoded by *PTEN* (phosphatase and tensin homolog; PTEN) is a known tumour suppressor and a critical inhibitor of the phosphatidylinositol (3,4,5)-trisphosphate (PIP3) branch of insulin signalling. On this basis, individuals would be expected to display improved insulin sensitivity, with a concomitant increase in cell growth and metabolism. Indeed, a recent study found individuals with Cowden’s syndrome to be profoundly insulin sensitive, even in the face of obesity [55]. This provides a dramatic example of the sometimes overlapping effects of intracellular signalling pathways involved in the regulation of metabolic and cell cycle-related processes.

### Mendelian randomisation as a tool for predicting adverse effects of risk factor modulation

Among studies within genetic epidemiology, a subset are based on a particular design known as Mendelian randomisation. The aim of Mendelian randomisation studies is to establish causal relationships between an environmental exposure and disease status [56]. More specifically, genetic variants are used as proxies for a modifiable exposure, which in turn may influence the outcome phenotype. As for other genetic association studies, the Mendelian randomisation design rests on the assumptions that genetic variants are fixed in time (not prone to reverse causation) and subject to independent assortment at conception (hence, more likely to produce unbiased estimates of a causal effect). In addition, Mendelian randomisation studies require that the selected variants influence disease status exclusively through the exposure of interest, and that they are not in linkage disequilibrium with any variants that could confound results [57]. If these conditions are satisfied, the paradigm can provide a powerful tool for causal inference without many of the confounding influences of conventional observational epidemiology. Most obviously, Mendelian randomisation studies can be used to define the role of environmental influences in disease aetiology, and thereby determine behavioural or molecular traits that can be modified to minimise risk.

Within a framework of drug target validation, Mendelian randomisation can be a useful strategy for exploring possible adverse effects of a proposed treatment. Unlike conventional GWAS/PheWAS, which seek to predict the side effects of drugs that modify a particular target, the aim of Mendelian randomisation is in doing so for any intervention that targets a particular risk factor. Recently, for example, Mendelian randomisation studies were used to delineate a clinically relevant link between treatments for cardiovascular disease and type 2 diabetes risk [58, 59]. Alleles in the genes *PCSK9* and *HMGCR*, known to predispose individuals to lower plasma LDL-cholesterol, were associated with the expected protective effect against cardiovascular events, but also with an inverse effect on type 2 diabetes risk. As the variants have no (known) pleiotropic effects, the results indicate a causal role of reduced LDL-cholesterol in type 2 diabetes susceptibility (among individuals that already have impaired glucose tolerance). Thus, the findings not only have implications for our understanding of current therapies targeting *HMGCR* (statins) and *PCSK9* (proprotein convertase subtilisin/kexin type 9 [PCSK9] inhibitors) but they also show that the same undesirable effect may turn out to be a general feature of any treatment that lowers LDL-cholesterol.

## Finding therapeutic indications based on pharmacogenomics and precision medicine

When balancing the expected effects of a drug candidate (both adverse and beneficial) any therapeutic hypothesis should be formulated in the context of an intended target population. Since not all individuals will benefit equally from a given treatment, identifying the most appropriate indication is critical to success in clinical trials. Clearly, the genetic associations identified during target discovery can be used to immediately propose broad indications for a candidate drug. By extension, PheWAS data can be used to search a larger phenotype space for any association that indicates a likely therapeutic or adverse effect.

The application of this principle extends beyond novel therapeutics and could be a powerful method for repositioning existing drugs for new indications [60]. One report overlapped known drug mechanisms with GWAS associations for each target [61]. Interestingly, around 40% of the associated traits matched the corresponding drug indication (e.g. the use of statins for hypercholesterolaemia is accurately predicted by *HMGCR* variants that are associated with LDL-cholesterol). Though this type of analysis will necessarily be limited to studying drugs that have known targets harbouring genetic variants associated with disease, the substantial overlap provides a validation of the approach and adds confidence to those indications corroborated by genetic evidence [61–63]. Still, more than half of the studied targets were associated with a different GWAS trait from that suggested by the indication for the drug. Some of these are likely a consequence of methodological limitations (e.g. difficulties translating GWAS signals), but the mismatches also highlight examples with additional supporting evidence. These represent plausible drug-repositioning opportunities.

### Pharmacogenomics: application in monogenic vs polygenic diabetes

The indications proposed by genetic associations are generally broad phenotypic labels. Within the field of type 2 diabetes, the heterogeneous nature of the disorder is often alluded to, sometimes with the implication that genetics could be used to inform more precise diagnostic categories. If clinically meaningful subtypes did exist, such diagnostic labels could likely improve treatment efficacy. Certainly, there are individuals carrying mutations with high, if not complete penetrance in specific genes. These individuals may either suffer from a monogenic form of diabetes or exist somewhere on the spectrum between complex type 2 diabetes and a Mendelian disorder [10, 15, 64]. Since disease progression in such individuals is determined by perturbations in a very limited number of pathways, genetic testing could in theory enable precision medicine.

Proof of concept has been provided by a life-changing treatment for individuals with permanent neonatal diabetes mellitus (PNDM). Genetic studies on PNDM has led to the realisation that a subset of individuals harbour mutations in the genes encoding the K_ATP_ channel [65]. Similar to the type 2 diabetes risk haplotype at this locus, the mutations were found to promote opening of the channel, suggesting that sulfonylureas could provide a disease-modifying therapy. This was confirmed in follow-up studies that demonstrated sustained efficacy in individuals with PNDM [66, 67]. Remarkably, most participants were able to discontinue insulin treatment, switching to oral therapy with improved metabolic control. Sulfonylureas have also been successful in disease management for certain forms of MODY. It was found that individuals with MODY carrying mutations in the *HNF1A* or *HNF4A* genes are sensitive to low-dose sulfonylureas, though the mechanism is incompletely understood [68–70]. The examples above, all of which are diseases with a defined genetic aetiology, provide compelling demonstrations that taking a pharmacogenetics approach can improve quality of life.

An interesting question pertains to whether such pharmacogenomic principles can be generalised to more complex forms of diabetes. In other words, can genetic testing identify subgroups of individuals with type 2 diabetes that are more likely to benefit from particular treatments than others? This would likely be the case if the underlying reality of diabetes was a collection of distinct subtypes, each dominated by defects in different pathways. As mentioned, it is clear that some individuals with type 2 diabetes do carry genetic variants with intermediate to high effect sizes that may be suggestive of increased sensitivity to drugs targeting the particular pathways affected. However, available evidence from genetic studies has shown that such individuals are in the minority and that the bulk of the genetic susceptibility for type 2 diabetes is carried by a very large number of common variants, each with small effect sizes. Equally, non-genetic factors, though less well understood, appear to be characterised by pervasive environmental perturbations. Individual risk profiles are thus dominated by exposures that are mostly common and widely shared, arguing against a model for disease architecture based on a set of distinct pathologies.

Emerging from the notion that existing disease models may be poorly suited for our current understanding of diabetes, an alternative taxonomy has recently been proposed [13, 71]; referred to as the ‘palette’ model (as opposed to a subtype-oriented model), it posits that diabetes is caused by a large number of small perturbations (environmental and genetic) across the component pathways of disease (e.g. beta cell function, insulin sensitivity, autoimmunity). Individually, the phenotypic impact of each perturbation is limited, but in aggregate will push a person on a path away from metabolic homeostasis. By analogy to colours combined in different hues and saturation, the palette taxonomy proposes an unlimited spectrum of disease manifestations. Monogenic and autoimmune forms of diabetes are represented in the extremes of this continuum [72]. It is thus an implication of this model that the majority of individuals are not dominated by defects in single or few processes [71]. These individuals cannot meaningfully be categorised into subtypes and, thus, attempts at delivering precision medicine will be challenging. It may be that biomarkers for specific processes can be used to glean insights into the pathways that are driving disease progression at any given time [71, 73]. As more process-modulating therapies become available, these could be used to encourage individuals along an appropriate trajectory, towards health. In the near future, however, targeting people with high-impact mutations (those at the extremes of the diabetes spectrum) are likely to be a more tractable aim for precision medicine.

## Experimental challenges for drug target validation using human genetics

A key aspect of translating GWAS signals into target validation naturally centres on the identification of the causal genes (or ‘effector transcripts’) driving disease susceptibility (Fig. 1). Despite advances in broadly understanding molecular and regulatory mechanisms involved in type 2 diabetes pathogenesis, progress on individual loci has been slow. A minority of the >100 independent association signals for disease risk have produced a single high-confidence candidate gene. As a result, the therapeutic value of GWAS for target discovery is still limited by this experimental bottleneck, especially since follow-up studies have tended to focus on the ‘low-hanging fruits’ supported by existing lines of evidence [74]. In the last few years, a number of different approaches have been taken to tackle this issue, providing complementary lines of evidence to enhance our understanding of causal mechanisms. The methods used for identifying effector transcripts broadly fall into three categories:

1. **The identification of coding risk variants to directly pinpoint effector transcripts** This approach has been facilitated by a recent shift in the attention of GWAS efforts towards low frequency and rare variants with higher penetrance [13, 75]. Even in regions with existing regulatory variants, coding variants can be used to direct experimental efforts towards particular candidates. This is illustrated by the *G6PC2/ABCC11* locus, which contained two strong candidate causal genes near a non-coding association signal identified for fasting glucose (an intermediate trait for type 2 diabetes susceptibility) [76, 77]. A more recent effort to map coding variation for glycaemic control found coding variants within the *G6PC2* gene [78, 79]. Follow-up experimental studies have since explored the effect of the variants to show a functional impact on the encoded glucose-6-phosphatase subunit [78]. An added benefit of finding causal variants in coding regions is the offer of a more straight-forward interpretation for therapeutic strategies. Non-coding variation is subject to the context-dependent activity of cis-regulation and the effects can be restricted to specific tissues or developmental stages [80]. As a consequence, drugs that target the affected gene could cause unexpected adverse effects by producing a more global phenotype. Though coding variants can also be subject to such context-dependency (e.g. through tissue-specific isoforms), the effector transcripts are often affected more widely [80, 81].
2. **Integration of genetic and genomic data to establish direct links between regulatory variation, genomic annotation and regional genes** One powerful approach within this category attempts to identify variants that affect the expression level of nearby genes, so-called cis-expression quantitative trait loci (cis-eQTLs). In cases where the association signal overlaps a cis-eQTL in a disease-relevant tissue, this can reveal both the target gene and the direction of effect for the risk variant. While many cis-eQTLs are shared across tissues, others appear to show more restricted effects that are specific to one or more tissues [82]. Since physiological characterisations of carriers of type 2 diabetes risk variants have implied a central role for islet dysfunction in disease susceptibility, several cis-eQTL studies have focused on pancreatic islets [83, 84]. Though the power to detect associations has been limited by the availability of islets from donors, the approach has successfully highlighted candidate effector transcripts with previously unknown roles in disease pathogenesis [85, 86]; this is the case for the poorly characterised *ZMIZ1* gene that was identified in a recent study [86]. In vitro work subsequently confirmed a role for *ZMIZ1* in islet function following functional studies.
  Intersecting human genetics with genomic annotation can also be used to define common regulatory themes that underlie causal mechanisms at multiple loci. A recent study, for example, demonstrated an enrichment of islet and liver binding sites for the forkhead box protein A2 (FOXA2) transcription factor among type 2 diabetes association signals [87]. These results suggest a shared role of FOXA2 across a subset of risk loci and highlight the potential to identify specific causal variants. In one instance, at the *MTNR1B* locus, where the association signal has been collapsed to a single variant through fine-mapping, the FOXA2 binding event was shown to be a marker for binding of another transcription factor, neurogenic differentiation 1 (NEUROD1). It was found that the risk allele creates a NEUROD1 binding site, leading to increased expression of *MTNR1B* in beta cells. This is in line with a cis-eQTL that was previously identified for this variant in islets, and adds support to a mechanism for this non-coding risk allele being mediated via elevated melatonin receptor 1B (MTRN1B; encoded by *MTNR1B*) activity [86, 88].
  Interestingly, a different direction of effect for the *MTRN1B* gene has been proposed by coding loss-of-function variants, which have also been associated with elevated risk of type 2 diabetes [89]. One potential explanation is suggested by the observation that the regulatory variant appears to exhibit tissue-specificity in its activity [87]. It is thus possible that the discrepancy between coding and non-coding variants could reflect differences between global and local roles of *MTRN1B*. Other explanations are possible and it remains to be seen whether increased *MTNR1B* transcript levels translate into higher protein expression. MTNR1B, a G-protein-coupled receptor, has received considerable attention as a potentially ‘druggable’ target. Addressing the inconsistencies in genetic data will thus provide insights into the suitability of MTNR1B as a drug target and inform any potential therapeutic strategies.

3. **Indirect prioritisation of genes based on known biology** Last among the methods for identifying causal mechanisms, a third category aims to indirectly prioritise genes based on known biology. For instance, a number of type 2 diabetes loci harbour genes implicated in monogenic forms of diabetes. Given the overlapping aetiologies between the diseases, monogenic diabetes genes can also be prioritised as causal for complex diabetes. Though this type of evidence is circumstantial, it could be a useful method for limiting the search space of genes to be studied. However, the number of loci for which current evidence favours one candidate over others is limiting and tends to be biased towards previously studied genes. One recently developed method aimed to sidestep this limitation by performing high-throughput functional characterisation of positional candidates for type 2 diabetes GWAS signals [90]. The screen successfully replicated known mechanisms of beta cell dysfunction and pointed to several unknown candidate causal genes. While any such study will be limited to a particular cell state, focusing on those tissues with high expected relevance to disease are likely to be most informative. Emerging genetic tools, such as clustered regularly interspaced short palindromic repeats **(**CRISPR)/CRISPR associated protein 9 (Cas9) and induced pluripotent stem cells, will make surveying a multitude of relevant phenotypes across tissue types and developmental stages an increasingly tractable goal.

## Conclusions

Available models for preclinical target validation have limited ability to assess causal relationships with direct relevance to humans. Advances in genetics and genomics hold the promise to bring down the cost of industry research and development pipelines by complementing these approaches. Through genotype–phenotype associations, ‘experiments of nature’ can, in principle, facilitate drug target validation. It is still too early to assess the impact of GWAS findings on prospective target discovery for diabetes treatment but the genes identified to date have successfully predicted known therapeutic mechanisms. These encouraging findings suggest that translating uncharacterised loci into pathophysiological mechanisms could point to novel drug targets. Increasingly, the uncovered therapeutic mechanisms may enable modes of precision medicine in diabetes for individuals with moderate- to high-penetrance mutations. For more common forms of diabetes, the extent to which pharmacogenetics will prove a relevant paradigm is still uncertain. Recent genetic insights have argued against a subtype-oriented taxonomy of disease, and more precise indications for type 2 diabetes therapies may not be a realistic target for the near future. Even so, human genetics could pave the way for new disease-modifying treatments that can benefit both common and rare forms of diabetes.

## Electronic supplementary material

ESM 1 (PPTX 169 kb)

## Abbreviations

Cis-eQTL: Cis-expression quantitative trait locus
FOXA2: Forkhead box protein A2
GKRP: Glucokinase regulatory protein
GWAS: Genome-wide association study
K_ATP_: ATP-sensitive potassium channel
MTRN1B: Melatonin receptor 1B
NEUROD1: Neurogenic differentiation 1
PheWAS: Phenome-wide association study
PNDM: Permanent neonatal diabetes mellitus
PPARγ: Peroxisome proliferator-activated receptor γ
SGLT2: Sodium–glucose cotransporter 2
TZD: Thiazolidinediones
ZnT8: Zinc transporter 8

## References

[1] IDF (2015) International diabetes federation diabetes atlas, 7th edn. International Diabetes Federation, Belgium. Available from http://www.diabetesatlas.org. Accessed 1 March 2017

[2] DiMasi JA, Feldman L, Seckler A, Wilson A (2010). Trends in risks associated with new drug development: success rates for investigational drugs. Clin Pharmacol Ther.

[3] DiMasi JA, Grabowski HG, Hansen RW (2016). Innovation in the pharmaceutical industry: new estimates of R&D costs. J Health Econ.

[4] Arrowsmith J, Miller P (2013). Trial watch: phase II and phase III attrition rates 2011-2012. Nat Rev Drug Discov.

[5] Cook D, Brown D, Alexander R (2014). Lessons learned from the fate of AstraZeneca's drug pipeline: a five-dimensional framework. Nat Rev Drug Discov.

[6] Wehling M (2009). Assessing the translatability of drug projects: what needs to be scored to predict success?. Nat Rev Drug Discov.

[7] Plenge RM, Scolnick EM, Altshuler D (2013). Validating therapeutic targets through human genetics. Nat Rev Drug Discov.

[8] Barrett JC, Dunham I, Birney E (2015). Using human genetics to make new medicines. Nat Rev Genet.

[9] Hindorff LA, Sethupathy P, Junkins HA (2009). Potential etiologic and functional implications of genome-wide association loci for human diseases and traits. Proc Natl Acad Sci U S A.

[10] MacArthur DG, Manolio TA, Dimmock DP (2014). Guidelines for investigating causality of sequence variants in human disease. Nature.

[11] Kryukov GV, Pennacchio LA, Sunyaev SR (2007). Most rare missense alleles are deleterious in humans: implications for complex disease and association studies. Am J Hum Genet.

[12] Goldstein DB, Allen A, Keebler J (2013). Sequencing studies in human genetics: design and interpretation. Nat Rev Genet.

[13] Fuchsberger C, Flannick J, Teslovich TM (2016). The genetic architecture of type 2 diabetes. Nature.

[14] Zuk O, Schaffner SF, Samocha K (2014). Searching for missing heritability: designing rare variant association studies. Proc Natl Acad Sci U S A.

[15] Flannick J, Beer NL, Bick AG (2013). Assessing the phenotypic effects in the general population of rare variants in genes for a dominant Mendelian form of diabetes. Nat Genet.

[16] Begg CB (2002). On the use of familial aggregation in population-based case probands for calculating penetrance. J Natl Cancer Inst.

[17] Zhou K, Pedersen HK, Dawed AY, Pearson ER (2016). Pharmacogenomics in diabetes mellitus: insights into drug action and drug discovery. Nat Rev Endocrinol.

[18] Price AL, Spencer CC, Donnelly P (2015). Progress and promise in understanding the genetic basis of common diseases. Proc R Soc B.

[19] Nelson MR, Tipney H, Painter JL (2015). The support of human genetic evidence for approved drug indications. Nat Genet.

[20] Hauner H (2002) The mode of action of thiazolidinediones. Diabetes Metab Res Rev 18(Suppl 2):S10–S15 10.1002/dmrr.249 11921433

[21] Altshuler D, Hirschhorn JN, Klannemark M (2000). The common PPARgamma Pro12Ala polymorphism is associated with decreased risk of type 2 diabetes. Nat Genet.

[22] Deeb SS, Fajas L, Nemoto M (1998). A Pro12Ala substitution in PPARgamma2 associated with decreased receptor activity, lower body mass index and improved insulin sensitivity. Nat Genet.

[23] Yen CJ, Beamer BA, Negri C (1997). Molecular scanning of the human peroxisome proliferator activated receptor gamma (hPPAR gamma) gene in diabetic Caucasians: identification of a Pro12Ala PPAR gamma 2 missense mutation. Biochem Biophys Res Commun.

[24] Majithia AR, Flannick J, Shahinian P (2014). Rare variants in PPARG with decreased activity in adipocyte differentiation are associated with increased risk of type 2 diabetes. Proc Natl Acad Sci U S A.

[25] Mahajan A (2014). Genome-wide trans-ancestry meta-analysis provides insight into the genetic architecture of type 2 diabetes susceptibility. Nat Genet.

[26] Janbon M, Chaptal J, Vedel A, Schaap J (1942). Accidents hypoglycémiques graves par un sulfamidothiodiazol (le VK 57 ou 2254 RP). Montp Med.

[27] Gloyn AL, Weedon MN, Owen KR (2003). Large-scale association studies of variants in genes encoding the pancreatic beta-cell KATP channel subunits Kir6.2 (KCNJ11) and SUR1 (ABCC8) confirm that the KCNJ11 E23K variant is associated with type 2 diabetes. Diabetes.

[28] Hani EH, Boutin P, Durand E (1998). Missense mutations in the pancreatic islet beta cell inwardly rectifying K+ channel gene (*KIR6.2/BIR*): a meta-analysis suggests a role in the polygenic basis of type II diabetes mellitus in Caucasians. Diabetologia.

[29] Gloyn AL, Hashim Y, Ashcroft SJ, Ashfield R, Wiltshire S, Turner RC (2001). Association studies of variants in promoter and coding regions of beta-cell ATP-sensitive K-channel genes SUR1 and Kir6.2 with type 2 diabetes mellitus (UKPDS 53). Diabet Med.

[30] Hamming KS, Soliman D, Matemisz LC (2009). Coexpression of the type 2 diabetes susceptibility gene variants KCNJ11 E23K and ABCC8 S1369A alter the ATP and sulfonylurea sensitivities of the ATP-sensitive K(+) channel. Diabetes.

[31] Proks P, Reimann F, Green N, Gribble F, Ashcroft F (2002) Sulfonylurea stimulation of insulin secretion. Diabetes 51(Suppl 3):S368–S376 10.2337/diabetes.51.2007.s368 12475777

[32] Sladek R, Rocheleau G, Rung J (2007). A genome-wide association study identifies novel risk loci for type 2 diabetes. Nature.

[33] Rutter GA, Chimienti F (2015). *SLC30A8* mutations in type 2 diabetes. Diabetologia.

[34] Flannick J, Thorleifsson G, Beer NL, Jacobs SB (2014). Loss-of-function mutations in *SLC30A8* protect against type 2 diabetes. Nat Genet.

[35] Bush WS, Oetjens MT, Crawford DC (2016). Unravelling the human genome-phenome relationship using phenome-wide association studies. Nat Rev Genet.

[36] Shiota C, Coffey J, Grimsby J, Grippo JF, Magnuson MA (1999). Nuclear import of hepatic glucokinase depends upon glucokinase regulatory protein, whereas export is due to a nuclear export signal sequence in glucokinase. J Biol Chem.

[37] Dupuis J, Langenberg C, Prokopenko I (2010). New genetic loci implicated in fasting glucose homeostasis and their impact on type 2 diabetes risk. Nat Genet.

[38] Lloyd DJ, St Jean DJ, Kurzeja RJ (2013). Antidiabetic effects of glucokinase regulatory protein small-molecule disruptors. Nature.

[39] Matschinsky FM (2009). Assessing the potential of glucokinase activators in diabetes therapy. Nat Rev Drug Discov.

[40] Saxena R, Voight BF, Lyssenko V (2007). Genome-wide association analysis identifies loci for type 2 diabetes and triglyceride levels. Science.

[41] Orho-Melander M, Melander O, Guiducci C (2008). Common missense variant in the glucokinase regulatory protein gene is associated with increased plasma triglyceride and C-reactive protein but lower fasting glucose concentrations. Diabetes.

[42] Johansen CT, Wang J, Lanktree MB (2010). Excess of rare variants in genes identified by genome-wide association study of hypertriglyceridemia. Nat Genet.

[43] Beer NL, Tribble ND, McCulloch LJ (2009). The P446L variant in GCKR associated with fasting plasma glucose and triglyceride levels exerts its effect through increased glucokinase activity in liver. Hum Mol Genet.

[44] Rees MG, Wincovitch S, Schultz J (2012). Cellular characterisation of the *GCKR* P446L variant associated with type 2 diabetes risk. Diabetologia.

[45] Rees MG, Ng D, Ruppert S (2012). Correlation of rare coding variants in the gene encoding human glucokinase regulatory protein with phenotypic, cellular, and kinetic outcomes. J Clin Invest.

[46] Rees MG, Raimondo A, Wang J (2014). Inheritance of rare functional GCKR variants and their contribution to triglyceride levels in families. Hum Mol Genet.

[47] Meininger GE, Scott R, Alba M (2011). Effects of MK-0941, a novel glucokinase activator, on glycemic control in insulin-treated patients with type 2 diabetes. Diabetes Care.

[48] De Ceuninck F, Kargar C, Ilic C (2013). Small molecule glucokinase activators disturb lipid homeostasis and induce fatty liver in rodents: a warning for therapeutic applications in humans. Br J Pharmacol.

[49] Chao EC (2014). SGLT-2 inhibitors: a new mechanism for glycemic control. Clin Diabetes.

[50] Ehrenkranz JR, Lewis NG, Kahn CR, Roth J (2005). Phlorizin: a review. Diabetes Metab Res Rev.

[51] Kanai Y, Lee WS, You G, Brown D, Hediger MA (1994). The human kidney low affinity Na+/glucose cotransporter SGLT2. Delineation of the major renal reabsorptive mechanism for D-glucose. J Clin Invest.

[52] van den Heuvel LP, Assink K, Willemsen M, Monnens L (2002). Autosomal recessive renal glucosuria attributable to a mutation in the sodium glucose cotransporter (SGLT2). Hum Genet.

[53] Tancredi M, Rosengren A, Svensson AM (2015). Excess mortality among persons with type 2 diabetes. N Engl J Med.

[54] Marsh DJ, Dahia PL, Caron S (1998). Germline PTEN mutations in Cowden syndrome-like families. J Med Genet.

[55] Pal A, Barber TM, Van de Bunt M (2012). PTEN mutations as a cause of constitutive insulin sensitivity and obesity. N Engl J Med.

[56] Smith GD, Ebrahim S (2003). ‘Mendelian randomization’: can genetic epidemiology contribute to understanding environmental determinants of disease?. Int J Epidemiol.

[57] Smith GD, Ebrahim S (2004). Mendelian randomization: prospects, potentials, and limitations. Int J Epidemiol.

[58] Schmidt AF, Swerdlow DI, Holmes MV (2016). *PCSK9* genetic variants and risk of type 2 diabetes: a mendelian randomisation study. Lancet Diabetes Endocrinol.

[59] Ference BA, Robinson JG, Brook RD (2016). Variation in PCSK9 and HMGCR and risk of cardiovascular disease and diabetes. N Engl J Med.

[60] Rastegar-Mojarad M, Ye Z, Kolesar JM, Hebbring SJ, Lin SM (2015). Opportunities for drug repositioning from phenome-wide association studies. Nat Biotechnol.

[61] Sanseau P, Agarwal P, Barnes MR (2012). Use of genome-wide association studies for drug repositioning. Nat Biotechnol.

[62] Sanseau P, Agarwal P, Barnes MR (2013). Reply to rational drug repositioning by medical genetics. Nat Biotechnol.

[63] Wang ZY, Zhang HY (2013). Rational drug repositioning by medical genetics. Nat Biotechnol.

[64] Althari S, Gloyn AL (2015). When is it MODY? Challenges in the interpretation of sequence variants in MODY genes. Rev Diabet Stud.

[65] Gloyn AL, Pearson ER, Antcliff JF (2004). Activating mutations in the gene encoding the ATP-sensitive potassium-channel subunit Kir6.2 and permanent neonatal diabetes. N Engl J Med.

[66] Pearson ER, Flechtner I, Njolstad PR (2006). Switching from insulin to oral sulfonylureas in patients with diabetes due to Kir6.2 mutations. N Engl J Med.

[67] Sagen JV, Raeder H, Hathout E (2004). Permanent neonatal diabetes due to mutations in KCNJ11 encoding Kir6.2: patient characteristics and initial response to sulfonylurea therapy. Diabetes.

[68] Shepherd M, Pearson ER, Houghton J, Salt G, Ellard S, Hattersley AT (2003). No deterioration in glycemic control in HNF-1alpha maturity-onset diabetes of the young following transfer from long-term insulin to sulphonylureas. Diabetes Care.

[69] Pearson ER, Pruhova S, Tack CJ (2005). Molecular genetics and phenotypic characteristics of MODY caused by hepatocyte nuclear factor 4alpha mutations in a large European collection. Diabetologia.

[70] Pearson ER, Starkey BJ, Powell RJ, Gribble FM, Clark PM, Hattersley AT (2003). Genetic cause of hyperglycaemia and response to treatment in diabetes. Lancet.

[71] McCarthy MI (2017) Painting a new picture of personalised medicine for diabetes. Diabetologia 60:793–799 10.1007/s00125-017-4210-x PMC6518376 28175964

[72] Gale EAM (2006). Declassifying diabetes. Diabetologia.

[73] Franks PW, McCarthy MI (2016). Exposing the exposures responsible for type 2 diabetes and obesity. Science.

[74] Thomsen SK, Gloyn AL (2014). The pancreatic beta cell: recent insights from human genetics. Trends Endocrinol Metab.

[75] Steinthorsdottir V, Thorleifsson G, Sulem P (2014). Identification of low-frequency and rare sequence variants associated with elevated or reduced risk of type 2 diabetes. Nat Genet.

[76] Bouatia-Naji N, Rocheleau G, Van Lommel L (2008). A polymorphism within the G6PC2 gene is associated with fasting plasma glucose levels. Science.

[77] Chen WM, Erdos MR, Jackson AU (2008). Variations in the G6PC2/ABCB11 genomic region are associated with fasting glucose levels. J Clin Invest.

[78] Mahajan A, Sim X, Ng HJ (2015). Identification and functional characterization of G6PC2 coding variants influencing glycemic traits define an effector transcript at the G6PC2-ABCB11 locus. PLoS Genet.

[79] Wessel J, Chu AY, Willems SM (2015). Low-frequency and rare exome chip variants associate with fasting glucose and type 2 diabetes susceptibility. Nat Commun.

[80] Thomsen SK, McCarthy MI, Gloyn AL (2016). The importance of context: uncovering species- and tissue-specific effects of genetic risk variants for type 2 diabetes. Front Endocrinol.

[81] Moltke I, Grarup N, Jorgensen ME (2014). A common Greenlandic TBC1D4 variant confers muscle insulin resistance and type 2 diabetes. Nature.

[82] GTEx Consortium (2015). Human genomics. The genotype-tissue expression (GTEx) pilot analysis: multitissue gene regulation in humans. Science.

[83] Voight BF, Scott LJ, Steinthorsdottir V (2010). Twelve type 2 diabetes susceptibility loci identified through large-scale association analysis. Nat Genet.

[84] Dimas AS, Lagou V, Barker A (2014). Impact of type 2 diabetes susceptibility variants on quantitative glycemic traits reveals mechanistic heterogeneity. Diabetes.

[85] Fadista J, Vikman P, Laakso EO (2014). Global genomic and transcriptomic analysis of human pancreatic islets reveals novel genes influencing glucose metabolism. Proc Natl Acad Sci U S A.

[86] van de Bunt M, Manning Fox JE, Dai X (2015). Transcript expression data from human islets links regulatory signals from genome-wide association studies for type 2 diabetes and glycemic traits to their downstream effectors. PLoS Genet.

[87] Gaulton KJ, Ferreira T, Lee Y (2015). Genetic fine mapping and genomic annotation defines causal mechanisms at type 2 diabetes susceptibility loci. Nat Genet.

[88] Tuomi T, Nagorny CL, Singh P (2016). Increased melatonin signaling is a risk factor for type 2 diabetes. Cell Metab.

[89] Bonnefond A, Clement N, Fawcett K (2012). Rare MTNR1B variants impairing melatonin receptor 1B function contribute to type 2 diabetes. Nat Genet.

[90] Thomsen SK, Ceroni A, van de Bunt M (2016). Systematic functional characterization of candidate causal genes for type 2 diabetes risk variants. Diabetes.

