# Supplementary material for: Human genetics as a model for target validation: finding new therapies for diabetes
Source: Diabetologia. 2017 Apr 26;60(6):960–70. doi: 10.1007/s00125-017-4270-y (PMC5423999; doi:10.1007/s00125-017-4270-y)
Supplement: Supplementary file 1 — (PPTX 169 kb) [file 125_2017_4270_MOESM1_ESM.pptx]

## Slide 1
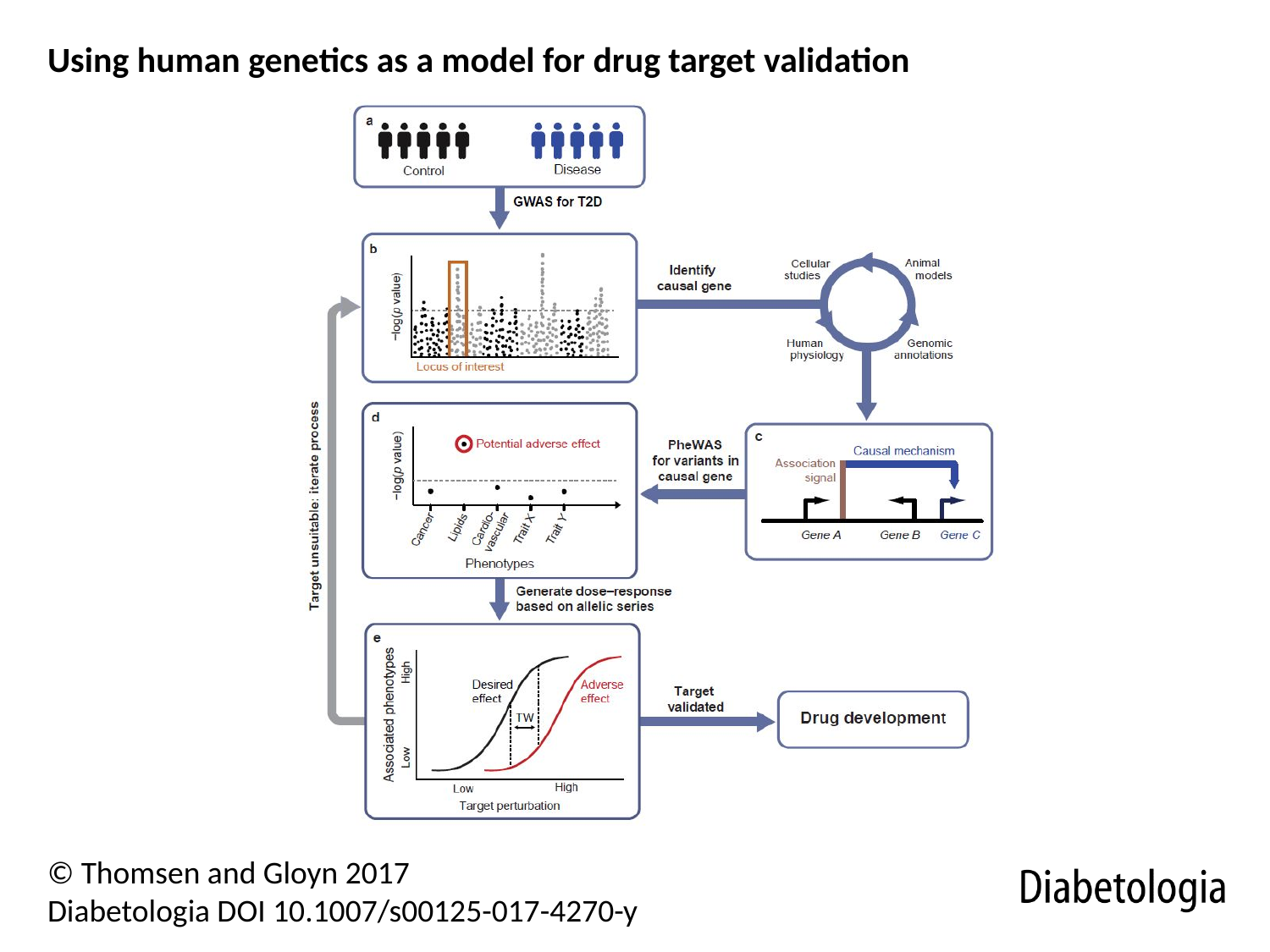

Using human genetics as a model for drug target validation
© Thomsen and Gloyn 2017
Diabetologia DOI 10.1007/s00125-017-4270-y
